# Bayesian regression model with application to a study of food insecurity in household level: a cross sectional study

**DOI:** 10.1186/s12889-021-10674-3

**Published:** 2021-03-30

**Authors:** Yenesew Fentahun Gebrie

**Affiliations:** Department of Statistics, Debre Markos University, P.O. Box 269, Debre Markos, Ethiopia

**Keywords:** Bayesian, Logistic regression, Household, Food insecurity

## Abstract

**Background:**

Food insecurity is a situation in which access to sufficient food is limited at times during the year by a lack of money and other resources. Even though several efforts were made to recover food security, still it is a critical social problem that needs immediate attention from policy and other decision makers especially in Ethiopia. The objective of the paper was to identify the significant predictors of food insecurity at household level in the given District.

**Method:**

A cross-sectional survey study was employed among 305 households selected using systematic random sampling technique. The data was collected using structured interviewer administrative questionnaire. Descriptive statistics was used to assess the prevalence of food insecurity status, and Bayesian estimation on binary logistic regression was used to identify the significant predictors of household food insecurity. Gibbs sampler algorithm was employed on Win BUGS software. Convergence of algorithm was assessed by using time series plot, density plot and auto correlation plot.

**Result:**

The prevalence of household food insecurity was 59% in the study District. From Bayesian estimation, the significant predictors of food insecurity were sex of household head, agro-ecological zone, loan status, access to agricultural training, age of household head, marital status of household head, family size, agricultural land size, tropical livestock unit, and soil fertility of agricultural land.

**Conclusion:**

The result shows that the households headed by male; who had own land, who land fertile soil, and those who took agricultural training were less likely to be food insecure. On the other hand, households with large family size, small farm land size and less tropical livestock unit were more likely to be food insecure. Hence, to increase food production and productivity of the farmers, proper attention should be given to improve soil fertility of agricultural land. Creating access to credit to households and providing them with agricultural training and family planning should be also emphasized.

**Supplementary Information:**

The online version contains supplementary material available at 10.1186/s12889-021-10674-3.

## Background

Food insecurity is a situation in which access to sufficient food is limited at times during the year because of lack of money and other resources. Additionally, it is social as well as economic problem, lack of food due to resource or other constraints, not voluntary fasting, or because of illness, or for other reasons [1, 2]. Food insecurity exists whenever there is limited or uncertain availability of nutritionally adequate and safe food or limited or uncertain ability to acquire acceptable food in socially acceptable ways [3, 4]. It also combines low food intake, variable access to food, and vulnerability for a livelihood strategy that generates adequate food in good times but is not resilient against shocks [5].

Around 795 million people worldwide do not have sufficient food to lead a healthy, active life. The vast majority of the world’s hungry people to live in developing countries; 12.9% of the population is undernourished. Asia is the continent with two-thirds of the total people suffering from starvation. In South Asia, the percentage has fallen in recent years, but it has slightly increased in Western Asia. Sub-Saharan Africa is the region with the highest prevalence of hunger that is, one in four people is starved [6]. Sub-Saharan Africa was hard hit by the global food crisis, due to short-term and long-term factors [7].

Food insecurity in Ethiopia stems directly from the reliance on undiversified livelihoods based on low-input and rained agriculture with low production. The outcomes correspond largely to chronic, cyclical, and transitory food insecurity, and in Ethiopia, they both are endemic. Structural factors leading to chronic food insecurity include poverty (as both cause and consequence), fragile natural resource base, weak institutions (notably markets, and land tenure), and unhelpful, or incoherent policies of government. Even though Ethiopia has made significant gains in education, an expanded system of health extensions and the fight against HIV / AIDS, food insecurity is still a public health problem.

A study carried out on the causes of household food insecurity in Wolayta, Southern Ethiopia revealed that age, family size, the number of livestock, input use, credit use and off-farm employment, self-consumption value, and dependence ratio had a significant effect on explaining the likelihood of a household being food insecure [8]. Besides, the study conducted in Damot Gale Woreda, Wolaita zone, southern Ethiopia, revealed that 71.6% of the households were food insecure. Households with large family size, non-educated, and aged household heads are more likely to be food insecure than those with smaller family size, educated and young household heads. Likewise, food insecurity resulted from households’ low land size and absence of livestock. Lack of confidence among households to overcome their food insecurity and their habit of borrowing money from informal rural lenders together with not using farm input are also significantly associated with food insecurity [9]. Another study conducted in Upper Blue-Nile, Ethiopia indicated that land size and family size were significantly correlated positively and negatively with household food security respectively [10]. Another study conducted in Tehuludere Woreda; South Wello Zone of Ethiopia showed that only 69.2% of the sample households were food insecure. The study put having large family size, small farm size, dependency attitude on food aid, poor wealth status (less than the sample mean TLU) and insecure land tenure perception as positive and significant factors that contributed to high food insecurity [11].

The debate on the causes of inconsistent food security among regions and communities have sparked increasingly divisive viewpoints across academic disciplines, and developmental thought over the last few decades which in turn result in a proliferation of demographic, economic, and political focus throughout the literature on food security [12]. Although there has been a good performance with high economic growth rates over the last decade, Ethiopia has not made significant progress on some of its major challenges, especially food security and employment for the growing youth population. Information is needed to properly target support, assess whether progress is being made, and develop appropriate interventions to assist those in need.

Food insecurity is a pressing issue of social and public health which varies in degree and effect among individuals and social groups. For this reason, understanding how food insecurity patterns appear across different demographic and socioeconomic issues is critical to meeting specific needs through the implementation of appropriate policy programs and other initiatives [13, 14]. Nowadays, Ethiopia has achieved strong economic growth, making it one of the highest performing economies in sub-Saharan Africa. Moreover, reducing food insecurity in the developing country like Ethiopia continues to be a major public policy challenge, and one that is complicated by lack of information on the location, severity, and causes of food insecurity. The current situation requires simultaneous and immediate scaling up of multi-dimensional life-saving and livelihood support along with investment in resilience building efforts in the most affected and at-risk areas. More than 80% of the population depends on agriculture for their food and income. Significant production losses have severely reduced the food security and purchasing power of households, forcing many to sell their remaining agricultural assets and to give up their livelihoods [15].

In the last two decades, Ethiopia has made substantial progress in poverty reduction. However, food insecurity is a threat to households as a result of events such as population growth, food prices and recurrent drought. Ethiopian government initiated a food security strategy built around: increasing the accessibility of food through domestic yield, ascertaining access to food for food deficient households, and strengthening institutional emergency response capability in 2004 [16]. However, food insecurity is a multidimensional concept experienced differently by different household types and population groups. Therefore, it is a complex issue that may not be fully captured by a one-dimensional item response model, especially as it will be used to track food insecurity over time, across different surveys, and for different sub-populations. Past literature on food insecurity has focused basically on children and single parent households. Moreover, the statistical model they employed was more of qualitative and cannot show the degree of severity of food insecurity.

Then, this study tries to assess the prevalence and significant predictors of food insecurity at a household level in Machakel district, East Gojjam Zone, Ethiopia using Bayesian binary logistic regression Model. Lastly, this study provides important information for households, policymakers and researchers.

## Methods and materials

### Study area and study design

This study was conducted in Machakel District which is located in East Gojjam Zone, Amhara region. Machakel is part of the East Gojjam Zone, which is bordered on the south by Debre Elias, on the northwest by the West Gojjam Zone, on the east by Sinan, and on the southeast by Gozamn. Based on the 2007 national census conducted by the Central Statistical Agency of Ethiopia, this District has a total population of 118,097, an increment of − 37.34% over the 1994 census.

A community based cross-sectional survey was conducted in Machakel District to investigate the degree of food insecurity, and its determinants in which the study population consisted of all households in the study area at the survey time.

### Data collection and variables

Primary data was used for this study, and they were collected through structured interviewer administrative questionnaire from selected households of Machakel District. The questionnaire was designed to gather qualitative and quantitative data. The questionnaire covered a range of topics including 18-item Core Food Security Module question series, socioeconomic and demographic variables meet objectives of the study.

Dependent variable of the study was household food security status (0 = food secure, 1 = food insecure). There is a strong rationale for measuring food insecurity at the household level. The United States department of Agriculture has been doing surveys of food insecurity using an 18-item Core Food Security Module question series. This module is recognized as the standard measure of food insecurity, and now it is used to measure food insecurity in virtually all national, state and local surveys.

For this study, important and common independent variables of food insecurity of households such as gender, age, religion, marital status and educational level of the household head, family size, and land size of the household were taken from different related literatures. Agro-ecological zone, saving habit, agricultural training, irrigation practice, slope of agricultural land, loan status, use of improved seed, use of fertilizer, land ownership, soil fertility of agricultural land, and Tropical livestock unit are also among the variables.

### Sampling technique and sample size determination

Multistage sampling technique was used for this particular study. In the first stage, the study Kebeles was stratified into two different strata to cover varying agro-climate. In the second stage, the two Kebeles were randomly selected. In the third stage, sample households were selected from each Kebele through systematic sampling method by taking the nth element of the sample frame. Sample size is determined by considering different situations such as objective of the research, design of the research, cost constraint, and degree of precision required. Based on these important ideas, the sample size of this study was determined by Cochran [17]:

$$ n=\frac{\frac{Z^2P\left(1-P\right)}{d^2}}{1+\frac{1}{N}\left(\frac{Z^2P\left(1-P\right)}{d^2}-1\right)}=\frac{\frac{(1.96)^2\ast (0.5)(0.5)}{(0.05)^2}}{1+\frac{1}{1480}\left(\frac{(1.96)^2\ast (0.5)(0.5)}{(0.05)^2}-1\right)}=305 $$

Where the variables represent their respective concepts as follow:

- n = sample size.
- N = total number of households in the selected Kebeles.
- d = margin of error 5% (0.05).
- P = is proportion of food insecurity = 0.5 with a 95% confidence level of standard normal distribution. Based on the above sample size formula, 305 sample households were obtained.

### Methods of data analysis

In this study, both descriptive statistics and Bayesian logistic regression model were used to meet the objective. Descriptive statistics was used to assess the prevalence of food insecurity and Bayesian logistic regression analysis was employed to identify the significant predictors of food insecurity at a household level.

### Bayesian logistic regression model

The logistic model is a family of regression model which is a special case of generalized linear model [18]. It is a statistical method for predicting the probability of an event, given a set of different covariates.

Bayesian logistic regression method is used to make inference about the parameters of the model, and this inference follows the common pattern for all other methods of analysis. This method considers the parameter of the model as random variables and data are considered as fixed, and the parameters have their prior distribution [19]. Bayesian estimation had better results than maximum likelihood estimation even under non-informative prior, especially for small samples on logistic regression model because it allows for probabilistic interpretations of the model coefficients [19–21]. The weakness of maximum likelihood estimation in small sample can be solved by Bayesian estimation as an alternative technique, and this estimation solves the challenge of assumption of classical approach since it is flexible [22].

This approach basically includes in expressing the data as a likelihood function, providing appropriate prior distribution to represent prior information on the variables, and then coming up with a posterior distribution of the model parameters, given data that are used in making inferences about the parameters.

### Likelihood function

For a sample of n observations, the joint distribution of Y_1_,..,Y_n_ is the product of n Bernoulli probabilities. The likelihood function used by Bayesian inference matches with the classical inference. The likelihood contribution from the i^th^ subject is binomial and the likelihood function is given by [23]:

1 $$ L\left({y}_i/\beta \right)=\prod \limits_{i=1}^n\left[{\left({P}_i\right)}^{y_i}{\left(1-{P}_i\right)}^{1-{y}_i}\right]=\prod \limits_{i=1}^n\left[{\left(\frac{e^{\beta_0+{\beta}_1{X}_{i1}+\cdots +{\beta}_k{X}_{ik}}}{1+{e}^{\beta_0+{\beta}_1{X}_{i1}+\cdots +{\beta}_{\mathrm{k}}{X}_{ik}}}\right)}^{y_i}{\left(1-\frac{e^{\beta_0+{\beta}_1{X}_{i1}+\cdots +{\beta}_k{X}_{ik}}}{1+{e}^{\beta_0+{\beta}_1{X}_{i1}+\cdots +{\beta}_k{X}_{ik}}}\right)}^{1-{y}_i}\right] $$

### Prior distribution

The special feature of Bayesian estimation is taking prior information on the parameters. This estimation of the model parameters requires the specification of a prior distribution for all the unknown parameters. For small samples, prior information can be critical because the maximum likelihood estimation on logistic regression model in small samples has significant bias and the serious inferential problems [19, 23]. In general, any prior distributions can be used depending on the available prior information.

The option can include informative prior distribution if something is known about the likely values of the unknown parameters or non-informative priors if either little is known about the coefficient values or if one wishes to see what the data themselves provide as inferences. Here, since there is no enough information or little prior knowledge about the value of parameters, we take non informative prior distribution.

The most common prior for logistic regression parameter is normal with the form: ${\beta}_j\sim N\left({\mu}_j,{\sigma}_j^2\right)$[23]. The prior distribution of logistic regression parameter is given by:

2 $$ P\left({\beta}_j\right)=\frac{1}{\sqrt{2\pi {\sigma}_j^2}}\ \mathit{\exp}\left\{\frac{-1}{2}{\left(\frac{\beta_j-{\mu}_j}{\sigma_j}\right)}^2\right\} $$

In Bayesian analysis, the precision is often specified instead of variance. The most common choice for *μ*_*j*_ is zero, and *σ*_*j*_ is usually chosen to be large enough to be considered as non-informative where common choices being in the range from *σ*_*j*_ = 10^2^to *σ*_*j*_ = 10^6^ [19].

### Posterior distribution

In Bayesian estimation, inference can be done from posterior distribution which is the combination of likelihood function and prior distribution [23].

The posterior distribution is proportional to product of the prior distribution over all parameters and likelihood function from the data which is given by:

3 $$ {\displaystyle \begin{array}{c}\pi \left(\beta / data\right)=\prod \limits_{i=1}^n\left[{\left(\frac{e^{\beta_0+{\beta}_1{X}_{i1}+\dots +{\beta}_k{X}_{ik}}}{1+{e}^{\beta_0+{\beta}_1{X}_{i1}+\dots +{\beta}_k{X}_{ik}}}\right)}^{y_i}{\left(1-\frac{e^{\beta_0+{\beta}_1{X}_{i1}+\dots +{\beta}_k{X}_{ik}}}{1+{e}^{\beta_0+{\beta}_1{X}_{i1}+\dots +{\beta}_k{X}_{ik}}}\right)}^{1-{y}_i}\right]\\ {}\ast {\prod}_{j=1}^k\frac{1}{\sqrt{2\pi {\sigma}_j^2}}\ \mathit{\exp}\left\{\frac{-1}{2}{\left(\frac{\beta_j-{\mu}_j}{\sigma_j}\right)}^2\right\}\end{array}} $$

The posterior distribution is complex function; hence, numerical methods are needed to obtain the marginal posterior distribution for each of the model parameters. Markov Chain Monte Carlo (MCMC) is the popular method in Bayesian estimation to obtain information from posterior distributions [24]. Gibbs sampling is the most common MCMC method to construct a Markov Chain for a target density, and it allows sampling from the full conditional distributions of each parameter, because sampling from the multivariate posterior distribution is not possible [25]. Gibbs sampling is a special case of Metropolis Hasting algorithm where the random value is always accepted (α = 1). Hence, the proposed move is accepted in all iterations [26]. Thus, the Gibbs sampler algorithm can be applied for this estimation using Win BUGS software to solve approximate the properties of the marginal posterior distributions for each parameter from the joint posterior distribution.

Steps of Gibbs sampling algorithm is given by:

1. Start the MC with the initial value*β*^*o*^, satisfying,${\beta}^o=\left({\beta}_1^o,{\beta}_2^o,\dots, {\beta}_k^o\right)$
2. Sample for i = 0,1,2, ...,n-1

4 $$ {\displaystyle \begin{array}{l}\mathrm{Generate}\kern0.5em {\beta}_1^{\left(i+1\right)} from\kern0.5em f\kern0.5em \left({\beta}_1/{\beta}_2^{(i)},{\beta}_3^{(i)}\dots, {\beta}_k^{(i)}\right)\\ {}\mathrm{Generate}\kern0.5em {\beta}_2^{\left(i+1\right)} from\kern0.5em f\kern0.5em \left({\beta}_2/{\beta}_1^{\left(i+1\right)},{\beta}_3^{(i)}\dots, {\beta}_k^{(i)}\right)\\ {}\vdots \\ {}\mathrm{Generate}\kern0.5em {\beta}_k^{\left(i+1\right)} from\kern0.5em f\left({\beta}_k/{\beta}_1^{\left(i+1\right)},{\beta}_2^{\left(i+1\right)}\dots, {\beta}_{k-1}^{\left(i+1\right)}\right)\end{array}} $$

3. Repeat step 2 until convergence

### Convergence of the algorithm

The convergence of MCMC algorithm is used to check whether the algorithm attained its target distribution. The Gibbs sampling algorithm is the most common Markov Chain Monte Carlo algorithms that converge to the target density as the number of iterations become large. Assessment of convergence can be done using the following plots: Time series plots which is most commonly used to assess convergence [27]; Gelman-Rubin statistic; density plots and autocorrelation plots. Accuracy of posterior estimates can be assessed based on the Markov Chain (MC) error. The simulations continue until the Monte Carlo error for each parameter of interest is less than about 5% of the sample standard deviation [28].

## Results

### General characteristics of the data

A total of 305 households from Machakel District were considered in this study. The prevalence of food insecurity status was considerable. The food insecurity status of households was determined from the 18-item Core Food Security Module question series designed by USDA. Out of the total sample, 59% of households were food insecure and the 41% were food secure (Table 1).

**Table 1:** Prevalence of food insecurity status of households

Household food insecurity status	Count	Percent
Food secure	125	41
Food insecure	180	59

From total households, majority (75.7%) were male headed, and 24.3% were female headed. Most households were married (73.8) whereas 26.2% were unmarried. Furthermore, nearly half (48.9) of the households were aged 45–64 and 27 (8.9%) of them were aged greater or equal to 65 year. Most of the households (92.1%) were Christian and 236 (77.4%) of the respondent can read and write. Almost half of the respondents (52.1%) had a family size 4–6, whereas 33.4 and 14.4% of them had a family size less than four and greater than six respectively (Table 2).

**Table 2:** Socio-demographic characteristics of the household head by food insecurity level

Variable	Category	Food secure	Food insecure	Total
		Count (%)	Count (%)	Count (%)
Sex of household head	Male	101 (80.8)	130 (72.2)	231 (75.7)
	Female	24 (19.2)	50 (27.8)	74 (24.3)
Age of household head	18–35	14 (11.2)	40 (22.2)	54 (17.7)
	36–44	34 (27.2)	41 (22.8)	75 (24.6)
	45–64	67 (53.6)	82 (45.6)	149 (48.9)
	> = 65	10 (8)	17 (9.4)	27 (8.9)
Religion of household head	Christian	112 (89.6)	169 (93.9)	281 (92.1)
	Muslim	13 (10.4)	11 (6.1)	24 (7.9)
Educational status household head	Can read and write	104 (83.2)	132 (73.3)	236 (77.4)
	Can’t read and write	21 (16.8)	48 (26.7)	69 (22.6)
Marital status household head	Married	98 (78.4)	127 (70.6)	225 (73.8)
	Unmarried	27 (21.6)	53 (29.4)	80 (26.2)
Family size of household	< 4	54 (43.2)	48 (26.7)	102 (33.4)
	4–6	61 (48.8)	98 (54.4)	159 (52.1)
	> = 7	10 (8)	34 (18.9)	44 (14.4)

In this study, environment, economy and practice related characteristics of households were considered. Regard to agro-ecological zone, about 248 (81.3%) of the sample had woina dega agro-ecological zone and 14.8% had kola agro-ecological zone. Most of the respondents i.e. around 253 (83%) of the households had a moderately (medium) sloped agricultural land while 7.2 and 9.8% of them had level and gentle sloped agricultural land respectively. Most of the households (79.3%) had no habit of credit service; only or nearly one-fifth of the respondents had habit of credit service. Regarding irrigation practice which has a contribution to reduce food insecurity, about 76.7% of the samples did not practice it. About 38.7% of households had less than or equal to one and half hectare of farm land, and 31.1% had two, and more hectares. The study revealed that 92.1% of the households use improved seed, and 89.2% of the sample used fertilizer. About 86.9% of the sample households have taken loan while 13.1% of them did not taken loan. Most households (92.5%) had the access to be trained by professionals on the agricultural extension package. Most of the respondents (88.2%) had fertile soil, and the rest 11.8% had infertile soil. Moreover, about 71.1% respondents had private farm land and 28.9% of them used rented farm land (Table 3).

**Table 3:** Agricultural environment, economic and practice related characteristics of household by food insecurity level

Variable	Category	Food secure	Food insecure	Total
		Count (%)	Count (%)	Count (%)
Agro-ecological zone	Kola	34 (27.2)	11 (6.1)	45 (14.8)
	Woinadega	87 (69.6)	161 (89.4)	248 (81.3)
	Dega	4 (3.2)	8 (4.4)	12 (3.9)
Slop of agricultural land	Level	12 (9.6)	10 (5.6)	22 (7.2)
	Medium	105 (84)	148 (82.2)	253 (83)
	Gentle slop	8 (6.4)	22 (12.2)	30 (9.8)
Saving habit	Yes	21 (16.8)	42 (23.3)	63 (20.7)
	No	104 (83.2)	138 (76.7)	242 (79.3)
Irrigation practice	Yes	23 (18.4)	48 (26.7)	71 (23.3)
	No	102 (81.6)	132 (73.3)	234 (76.7)
Land size in hectare	<=1.5	34 (27.2)	84 (46.7)	118 (38.7)
	1.6–2	33 (26.4)	59 (32.8)	92 (30.2)
	> 2	58 (46.4)	37 (20.6)	95 (31.1)
Have you taken Loan	Yes	6 (4.8)	34 (18.9)	40 (13.1)
	No	119 (95.2)	146 (81.1)	265 (86.9)
Tropical livestock unit	< 2.5	46 (36.8)	71 (39.4)	117 (38.4)
	> = 2.5	79 (63.2)	109 (60.6)	188 (61.6)
Use of improved seed	Yes	114 (91.2)	167 (92.8)	281 (92.1)
	No	11 (8.8)	13 (7.2)	24 (7.9)
Training by agricultural profession	Yes	111 (88.8)	171 (95)	282 (92.5)
	No	14 (11.2)	9 (5)	23 (7.5)
Soil fertility of agricultural land	Fertile	123 (98.4)	146 (81.1)	269 (88.2)
	Infertile	2 (1.6)	34 (18.9)	36 (11.8)
Use of fertilizer	Yes	111 (88.8)	161 (89.4)	272 (89.2)
	No	14 (11.2)	19 (10.6)	33 (10.8)
Land ownership	Private	81 (64.8)	136 (75.6)	217 (71.1)
	Rented	44 (35.2)	44 (24.4)	88 (28.9)

### Result of Bayesian logistic regression analysis

The main objective of this study was to identify the significant factors of food insecurity in Machakel district. Bayesian binary logit model was used to identify potential explanatory variables of household’s food insecurity, and Gibbs sampler algorithm was used on Win BUGS software. The Gibbs sampler algorithm was employed with 20,000 iterations in three, different chains that generate 45,000 samples from the posterior distribution at 5001 burn-in terms discarded. Based on the result of this estimation, sex of household head, age of household head, marital status of household head, family size, ecological zone, tropical livestock unit, loan status, access to agricultural training, land size, soil fertility and land ownership were significant predictors of food insecurity at α = 5% because the 95% credible interval of these variables does not include zero (at least one category). Based on the result, households with large family size, households headed by female, unmarried household (single, divorced and widowed) head and old household head were more likely to be food insecure than their counterpart. Based on the value of posterior mean, land size, land ownership (rented), tropical livestock unit, access to agricultural training (no) and loan status (no) had significant negative association with food insecurity. But soil infertility, family size (small) and woina dega ecological zone had significant positive relationship with food insecurity (Table 4)*.*

**Table 4:** Result of Bayesian logistic Regression Analysis of food insecurity of household level

Node	Category	Coefficient	SE	MC Error	95.0% CI ($\hat{\beta}$)	
					Lower	Upper
Constant		1.320	1.106	0.038	−0.773	3.518
Sex of household head	Female	1.467*	0.493	0.005	0.536	2.469
Religion	Muslim	−0.481	0.735	0.007	−1.952	0.931
Agro-ecological zone	Woinadega	1.764*	0.579	0.012	0.644	2.922
	Dega	1.463	0.992	0.011	− 0.451	3.449
Slope of agricultural land	Medium	−0.130	0.692	0.018	− 1.474	1.235
	Gentle slope	1.126	0.899	0.018	−0.609	2.942
Saving habit	No	−0.081	0.449	0.006	−0.976	0.791
Taken loan	No	−1.334*	0.648	0.015	−2.642	−0.134
Irrigation practice	No habit	0.032	0.438	0.006	−0.831	0.886
Use of improved Seed	No	1.422	0.825	0.008	−0.130	3.104
Land ownership	Rented	−0.864*	0.422	0.005	−1.695	−0.042
Access to training	No	−1.514*	0.757	0.007	−3.027	−0.066
Age of household head	36–44	−2.748*	0.587	0.009	−3.936	−1.645
	45–64	−2.572*	0.531	0.009	−3.64	−1.571
	> = 65	−1.784*	0.709	0.009	−3.209	−0.422
Fertilizer use	No	−1.158	0.636	0.006	−2.419	0.0683
Marital status	Others	1.025*	0.475	0.005	0.109	1.970
Education of household head	No read &write	0.724	0.464	0.005	−0.175	1.637
Family size of household	4–6	1.54*	0.452	0.007	0.669	2.447
	> = 7	2.938*	0.648	0.008	1.712	4.252
Land size in hectare	1.6–2	−0.096	0.417	0.004	−0.913	0.722
	> 2	−1.313*	0.439	0.006	−2.188	−0.457
TLU	> = 2.5	−0.859*	0.387	0.004	−1.633	−0.117
Soil fertility	Infertile	2.809*	0.939	0.006	1.216	4.895

First category = Reference category, * = significant at 5% level of significance

### Assessment of convergence and accuracy of the model

Assessment of convergence can be done using the time series plots, Gelman-Rubin statistic, density plots and autocorrelation plots. It is indicated in the given plots below that in any plot of significant predictors the convergence of the algorithm was attained (see figures on the Additional file 1).

## Discussion

In this study, the prevalence and significant predictors of food insecurity of household in the given District were identified. Bayesian logistic regression model was employed to identify the significant predictors using Gibbs sampler algorithm on Win BUGS software. Out of total samples, the prevalence of food insecurity of household was found to be 59% (Table 1). The algorithm was employed with 20,000 iterations in three different chains and 45,000 samples from the posterior distribution at 5001 burn-in terms were discarded.

According to this study, gender of the household was found to be the significant predictor for food insecurity. The household headed by female is more likely to be food insecure than the one headed by male and this variation may be due to the culture. This study encourages giving attention to female headed households to fill the variation. The result is similar with the study [29], and it is inconsistent with study of [8–10, 30–32] in Ethiopia.

The study indicated also that age of the household head to be significant predictors for household’s food insecurity status. Age of household head was negatively related with food insecurity which indicates those households headed with older age were less likely to be food insecure. It may due to having experience, and it is supported by other study conducted by [8, 9, 29, 31] and this idea is not supported by the following study [10, 30].

Based on the result, family size of the household was significantly and positively associated with food insecurity. Hence, household with large family size was more likely to be food insecure, this may be due to large household size uses more consumptions of food. The result encourages for education (awareness) about lower birth rates to reduce family size. This result is supported by similar findings of [8–11, 29, 31, 33] and this result is not similar with the study [30, 32].

As a result shown, marital status of the household head was found to be significant factor for food insecurity status. Currently married household head were less likely to be food insecure as compared with unmarried ones. The reality is also true because married household head can be supported by his/her wife/husband and this idea is consistent with similar finding [29].

The ecological zone was significant predictors for household food insecurity. Households living in Woina dega were less likely to be food insecure compared to living in Kola agro-ecological zone. The result is consistent with the finding of [10, 31, 33]. This may be since the variation of cereal crop in Woina dega and kola agro-ecological zone.

The result found that size of farm land was negatively and significantly associated with household food insecurity. This indicates those households who had lower land size were more likely to be food insecure as compared with counterpart and the result is consistent with the following findings [9, 10, 30, 31] whereas it is inconsistent with the study of [8].

As shown in the result, land ownership of the household was significant predictor for food insecurity status. Furthermore, households who rent farm land were more likely to be food insecure than those who had their land, and the result is supported by similar finding [29].

Tropical livestock unit of the household was also significant predictor for food insecurity status. Household who had more tropical livestock unit were less likely to be food insecure than those who had few livestock, and this idea is consistent with the findings of [8–11, 33].

According to the study, soil fertility of farm land was significantly associated with food insecurity. Household with fertile soil were more likely to be food secure. This may be due to the fact that farming on fertile soil will be able to increase agricultural productivity and the idea is inconsistent with other study [31]. This study encourages giving attention to healthy soil on soil management and awareness of soil productivity improvement.

Another significant factor for food insecurity status was loan status of the respondents. Households that do not taken a loan from any financial institution were more likely to be food insecure compared to households that take a loan. This indicates that the risk of being food insecure for households who do not take a loan was higher than households who take a loan, and this result also supported by the following studies [8, 9]. This may be due to the fact that households who take loan can increase household income to purchase livestock and input for agricultural activities.

## Conclusion

The objective of this study was to assess the prevalence of food insecurity and to identify its significant factors in Machakel District. Bayesian logistic regression model was used to identify the significant predictors of food insecurity. The study shows that 59% of the household were food insecure. From Bayesian estimation, the significant predictors of food insecurity were sex of household head, agr-ecological zone, loan status, access to agricultural training, age of household head, marital status of household head, family size of household, farm land size, tropical livestock unit, and soil fertility of farm land. The result revealed that household with large family size; less tropical livestock unit and small farm land size were more likely to be food insecurity. The household headed by male, married household head, who had their own land with fertile soil, and who take agricultural training were less likely to be food insecure. Similarly, taking loan and age of the household head were significantly associated with food insecurity. Since household food insecurity was highly considerable, proper attention should be given to increase food production and productivity of the households. The responsible body should create sufficient awareness on family planning to minimize family size. Agricultural sectors should give priority to give training on agricultural extension package and livestock sector development thereby minimizing food insecurity. Head of the household should keep their soil fertility and female household head should improve their competence. Last, further research is needed to identify other factors of food insecurity on each specific case because this idea is broad.

## Supplementary Information

**Additional file 1.**

## Data Availability

The primary data set collected from households and analyzed during the current study is available from the corresponding author.

## Abbreviations

CSA: Central Statistical Agency
CFSM: Core food security module
FAO: Food and Agriculture Organization
HH: Head of household
MDG: Millennium Development Goal
TLU: Tropical livestock unit
USDA: United state Department of Agriculture
WFS: World Food Summit
