# Supplementary material for: Bayesian regression model with application to a study of food insecurity in household level: a cross sectional study
Source: BMC Public Health. 2021 Mar 30;21:619. doi: 10.1186/s12889-021-10674-3 (PMC8008667; doi:10.1186/s12889-021-10674-3)
Supplement: Supplementary file 1 — Additional file 1. [file 12889_2021_10674_MOESM1_ESM.docx]

**Supplementary file (images)**

Different Plots to Assess the Convergence of Algorithm

1. **Time series plots for predictors**

1. **Gelman plots of predictors**

1. **Density plots for predictors**

1. **Autocorrelation plots for predictors**
